# Comparable Euploidy and Aneuploidy Patterns Between Utrogestan-Based Progesterone-Primed Ovarian Stimulation and GnRH Antagonist in PGT-M: A Retrospective Matched Cohort Study

**DOI:** 10.3390/biomedicines14071629

**Published:** 2026-07-20

**Authors:** Xiaolan Li, Shujing He, Yajie Chang, Pan Chen, Yanfang Wang, Xiaoyan Liang, Zhiqiang Zhang, Jingjie Li

**Affiliations:** 1 Reproductive Medicine Center, The Sixth Affiliated Hospital, Sun Yat-sen University, Guangzhou 510655, China; lixlan25@mail.sysu.edu.cn (X.L.); heshj8@mail.sysu.edu.cn (S.H.); changyj3@mail.sysu.edu.cn (Y.C.); wangyf255@mail.sysu.edu.cn (Y.W.); 2 Guangdong Engineering Technology Research Center of Fertility Preservation, Guangzhou 510655, China; 3 Biomedical Innovation Center, The Sixth Affiliated Hospital, Sun Yat-sen University, Guangzhou 510655, China; 4 Department of Pharmacy, The First Affiliated Hospital, Sun Yat-sen University, Guangzhou 510655, China; chenp73@mail.sysu.edu.cn

**Keywords:** progestin-primed ovarian stimulation protocol, Utrogestan, preimplantation genetic testing for monogenic disorders, aneuploidy, gonadotropin-releasing hormone antagonist

## Abstract

**Objectives:** This study compared controlled ovarian hyperstimulation (COH) outcomes and chromosomal euploidy results between the Utrogestan-based progesterone-primed ovarian stimulation (PPOS) and gonadotropin-releasing hormone antagonist (GnRH-Ant) protocols in preimplantation genetic testing for monogenic disorders (PGT-M) cycles, and assessed the efficacy and safety of the Utrogestan-based PPOS protocol. **Methods:** In this retrospective single-center cohort study, 176 PGT-M cycles managed with the Utrogestan-based PPOS protocol were compared with 176 GnRH-Ant cycles using 1:1 direct caliper matching without replacement. Embryos were classified as euploid, mosaic, aneuploid, or no-call according to next-generation sequencing results. The primary outcomes were euploidy rate and chromosomal aberration patterns; secondary outcomes included COH outcomes and pregnancy outcomes. **Results:** Baseline characteristics were comparable after matching. The total gonadotropin (Gn) dose was significantly lower in the Utrogestan-based PPOS group (2100 (1500–3000) vs. 2250 (1800–3000), *p =* 0.035). PGT analysis revealed comparable rates of euploidy, aneuploidy, mosaicism, and “No-call” between the Utrogestan-based PPOS (*n* = 586) and GnRH-Ant (n = 605) groups. The unadjusted difference in euploidy rate per MII oocyte between groups (18.98% vs. 16.36%, *p* = 0.045) was no longer statistically significant after multivariable adjustment (*p* = 0.194). In addition, patterns of aneuploidy and chromosomal involvement were similar between groups. The Utrogestan-based PPOS group showed numerically higher clinical pregnancy rate (70.67% vs. 63.22%) and live birth rate (62.67% vs. 51.72%), and a lower early abortion rate (5.66% vs. 12.73%), but none of these differences were statistically significant. **Conclusions:** Compared with the GnRH-Ant protocol, the Utrogestan-based PPOS protocol required a significantly lower total Gn dose while exhibiting comparable euploidy rate, analogous chromosomal abnormality spectrums, and pregnancy outcomes. The Utrogestan-based PPOS protocol may be a feasible alternative ovarian stimulation strategy in PGT-M cycles.

## 1. Introduction

Premature luteinizing hormone (LH) surge, which is caused by significantly elevated plasma estradiol concentrations resulting from the multi-follicular development [1], is a major cause for cycle cancelation during controlled ovarian hyperstimulation (COH). Over the past few decades, the prevention of a premature LH surge has mainly relied on the use of GnRH agonist (GnRH-a) and GnRH antagonist (GnRH-Ant) [2]. In 2015, Kuang et al. first reported that a progestin-primed ovarian stimulation (PPOS) protocol using medroxyprogesterone acetate (MPA) could effectively prevent premature LH surge [3]. Since then, lots of studies have declared that PPOS protocol can effectively prevent premature LH surge [4,5,6]. The use of oral progestins makes the PPOS protocol an attractive alternative in COH, especially in settings where a freeze-all strategy is planned, owing to its patient-friendly nature and cost-effectiveness.

Nevertheless, the efficacy and safety of PPOS protocols remain controversial. In the PPOS protocol, progesterone is administered from the early follicular phase until oocyte retrieval. Whether early follicular-phase exposure to supraphysiological progesterone levels and exogenous progestins affects embryonic developmental potential and chromosomal euploidy remains a major concern for both clinicians and patients. In 2018, our team reported that follicular fluid hormone levels and granulosa cell apoptosis were not affected under the PPOS protocol, suggesting no adverse impact on follicular development [7]. However, real-world evidence regarding COH outcomes and embryo aneuploidy following PPOS remains controversial [8,9,10,11,12].

Several progestins have been used in PPOS protocols, including medroxyprogesterone acetate (MPA), dydrogesterone, and micronized progesterone. From a reproductive safety perspective, these agents may not be identical. MPA has historically been contraindicated during pregnancy, and recent pharmacovigilance data have raised concerns regarding a possible association between dydrogesterone exposure and congenital abnormalities [13]. In contrast, Utrogestan is a formulation of natural micronized progesterone, which is structurally identical to endogenous progesterone and has been widely used in reproductive medicine, particularly for luteal-phase support and early pregnancy exposure. These features make Utrogestan a clinically relevant progestin option for PPOS protocols in our center. However, whether the differences in reproductive safety profiles among progestins translate into altered embryonic chromosomal status remains unknown. There is a paucity of reports regarding oral Utrogestan-based PPOS regimens on embryonic euploidy rates and chromosomal aberration patterns. Moreover, most previous studies investigating embryonic chromosomal aneuploidy have recruited populations undergoing PGT for aneuploidy (PGT-A) cycles [10,11,12]. This cohort exhibits high heterogeneity, as it includes patients with recurrent miscarriage, repeated implantation failure, or advanced age—conditions that are inherently associated with an increased incidence of embryonic chromosomal aneuploidy. In contrast, patients undergoing PGT for monogenic disorders (PGT-M) constitute a relatively homogeneous population with a lower prevalence of infertility-related risk factors. Thus, this population offers a suitable cohort for isolating the effect of the Utrogestan-based PPOS protocol on embryonic chromosomal status. Therefore, we conducted a retrospective analysis comparing chromosomal euploidy results and COH outcomes between the Utrogestan-based PPOS and GnRH-Ant protocols in PGT-M cycles to evaluate the efficacy and safety of the PPOS protocol.

## 2. Methods

### 2.1. Study Population

This retrospective cohort study evaluated the impact of ovarian stimulation protocols on embryonic chromosomal status in combined PGT-M cycles. Couples who underwent PGT-M at our reproductive medicine center between April 2019 and December 2025 were included. During the study period, 270 cycles were initiated using the GnRH-ant protocol and 531 cycles using the Utrogestan-based PPOS protocol. To minimize selection bias, 1:1 direct caliper matching without replacement was performed based on maternal age (±1 year), antral follicle count (AFC) (±2), and serum anti-Müllerian hormone (AMH) level (±0.5 ng/mL). This matching procedure yielded 176 matched cycles in each group for a comparative analysis between the two protocols. All couples had a confirmed indication for PGT-M based on pathogenic or likely pathogenic variants identified through prior genetic counseling and molecular diagnosis. Patients were excluded if they met any of the following criteria: (1) use of other protocols including long GnRH-agonist protocol, dydrogesterone-based PPOS, mild stimulation, luteal-phase stimulation, etc.; (2) use of oocyte donation or thawed embryos for biopsy; (3) cancelation of oocyte retrieval; (4) a history of recurrent miscarriage or repeated implantation failure.

This study was reviewed and approved by Ethics Committee of the Sixth Affiliated Hospital, Sun Yat-sen University, on 24 March 2025 (approval number: 2025ZSLYEC-151), and conducted in accordance with the Declaration of Helsinki for medical research. Written informed consent was obtained from all patients after they had received comprehensive counseling regarding infertility treatments and routine PGT procedures. The study was based on existing clinical and embryology laboratory records generated during routine clinical care. No prospective intervention or randomization was performed for research purposes. Data extraction, anonymization, and statistical analysis were conducted after ethics approval had been obtained.

### 2.2. Assisted Reproductive Technology Procedure

Individual COH protocols and initial dose of gonadotropin (Gn) were determined according to patient characteristics, such as age, body mass index (BMI), basal follicle-stimulating hormone (FSH), AMH, AFC, etc. In the PPOS group, patients received Gn, either recombinant FSH (Gonal-f^®^, Merck Serono, Darmstadt, Germany) or urinary FSH (Lishenbao^®^, Livzon Pharmaceutical Group Inc., Zhuhai, China), together with oral progesterone (Utrogestan, Besins Manufacturing Belgium, Brussels, Belgium) at a dose of 0.3 g/d, starting on day 2 or 3 of the menstrual cycle and continuing until oocyte retrieval. In the fixed GnRH-Ant protocol group, Gn was initiated on day 2 or 3 of the menstrual cycle, with a daily subcutaneous injection of 0.25 mg GnRH-Ant (Cetrotide, Merck Serono, Geneva, Switzerland) initiated on days 7–8 of the cycle. For both protocols, Gn doses were further adjusted according to ovarian response after 5 days of stimulation. When at least 2 to 3 follicles reached a diameter of 16–18 mm, 4000–10,000 IU of human chorionic gonadotropin (hCG) (Livzon Group, Zhuhai, China) was administered intramuscularly. Oocyte retrieval was conducted 34–38 h after hCG administration under transvaginal ultrasound guidance.

On the day of oocyte retrieval, semen samples were collected by masturbation and allowed to liquefy at room temperature before processing by density gradient centrifugation. Only metaphase II (MII) oocytes, identified by the presence of the first polar body, were selected for intracytoplasmic sperm injection. Fertilization was evaluated 16–19 h after insemination, and normal fertilization was defined as the presence of two pronuclei and two polar bodies. Embryos were further cultured in G1/G2 sequential media (Vitrolife, Gothenburg, Sweden) at 37 °C in incubators with 6% CO_2_ and monitored until days 5–6 after insemination. Embryo morphology was assessed on day 3 and day 5–6 after insemination. Blastocyst quality was evaluated using the Gardner grading system [14]. Approximately 5–10 trophectoderm cells were aspirated using standardized biopsy techniques, and all biopsied blastocysts were subsequently vitrified following the manufacturer’s protocol (VT101, Kitazato, Fuji, Shizuoka, Japan).

Following PGT-M analysis, patients underwent frozen-thawed embryo transfer (FET) if they had at least one euploid blastocyst. Endometrial preparation protocols for FET were determined based on each patient’s ovulatory status: ovulatory patients underwent natural-cycle preparation, whereas anovulatory patients received hormone replacement therapy. Embryo transfer was scheduled once the endometrial thickness reached ≥7 mm, and a single euploid embryo was transferred in all FET cycles.

### 2.3. PGT-M and PGT-A Procedures

Trophectoderm cells obtained by biopsy were transferred into RNase- and DNase-free polymerase chain reaction tubes preloaded with 5 μL of cell lysis buffer (XK043, Yikon Genomics, Suzhou, China). Whole-genome amplification was performed using the multiple annealing and looping-based amplification cycles technique. PGT-M was conducted using family-specific haplotype analysis based on informative single-nucleotide polymorphism (SNP) markers to determine the inheritance status of pathogenic variants. Concurrently, PGT-A was performed using next-generation sequencing on an Illumina platform (NextSeq 550, Illumina, San Diego, CA, USA) to assess genome-wide copy-number variations. Based on the NGS results, embryos were classified as euploid, mosaic (intermediate copy number suggestive of mosaicism), or aneuploid according to PGDIS recommendations [15]. Aneuploid embryos were further categorized as simple, double, or complex aneuploidy according to the number of affected chromosomes.

### 2.4. Quality Control (QC) and Contamination Assessment

Sequencing data were evaluated using predefined QC metrics. The median absolute pairwise difference (MAPD) was calculated as the median of absolute differences between adjacent bin values (MAPD = median(∣X_(i+1)_ − X_i_∣)), reflecting local copy-number variability. Bin coefficient of variation (CV) was defined as the median coefficient of variation in read ratios across all bins on each chromosome (CV = standard deviation (SD)/mean). Samples that failed to meet QC thresholds were excluded from further analysis and classified as no-call. Parental contamination was assessed via B-allele frequency analysis of SNP loci.

### 2.5. Study Outcomes

The primary outcomes were chromosomal euploidy results, including euploidy rate and chromosomal aberration patterns (simple, double, or complex). Secondary outcomes included the duration of Gn stimulation, total Gn dose, formation rate of blastocysts available for biopsy, formation rate of blastocysts available for biopsy per MII oocyte, and pregnancy outcomes. Biochemical pregnancy was defined as serum β-human chorionic gonadotropin (β-hCG) level ≥ 25 U/L on days 10–12 after embryo transfer. Clinical pregnancy referred to the detection of a gestational sac via transvaginal ultrasonography, and live birth was defined as the complete expulsion or extraction of a fetus from its mother, followed by breathing or other evidence of life.

### 2.6. Statistics

After 1:1 direct caliper matching, all comparative analyses were performed with consideration of the matched design. Covariate balance before and after matching was assessed using standardized mean differences (SMDs), with an absolute SMD < 0.10 indicating adequate balance. Continuous variables were expressed as mean (SD) for normally distributed data and median (interquartile range [IQR]) for non-normally distributed data. Categorical data were presented as counts (percentages). Paired Student’s *t*-tests or Wilcoxon signed-rank tests were used for continuous variables, whereas McNemar’s tests or Bowker’s tests were used for paired categorical variables, as appropriate. Proportional outcomes were compared using generalized estimating equations (GEE) with a binomial distribution and logit link, accounting for within-pair correlation. The distribution of PGT-A outcomes was assessed using cluster-robust multinomial logistic regression (implemented in Stata 17.0). For outcomes with expected cell counts < 5 in either group, formal hypothesis testing was not performed; only descriptive statistics are reported. To address residual confounding, multivariable analyses of euploidy rates were performed using GEE (binomial, logit link). Missing data were minimal (missing rate < 5% for all variables) and were handled by complete-case analysis. Statistical analyses were performed with SPSS 25.0, and statistical significance was defined as *p* < 0.05. Post hoc power calculations were conducted using G*Power 3.1 (two-tailed α = 0.05) based on the observed proportions and sample sizes.

## 3. Results

### 3.1. Baseline Characteristics

A total of 352 cycles were obtained after 1:1 direct caliper matching, comprising 176 cycles in each group. As presented in Table 1, all matching variables were well balanced between the two groups, with absolute SMD values < 0.10. The demographic parameters and baseline clinical parameters of the eligible subjects were summarized in Table 1. No significant differences were found between the Utrogestan-based PPOS and GnRH-Ant groups in maternal age, paternal age, AMH, AFC, BMI, and basal hormone s. Specifically, the mean maternal age was 31.57 ± 3.77 years in the Utrogestan-based PPOS group and 31.45 ± 3.79 years in the GnRH-Ant group, respectively. Overall, the primary indications for PGT-M in both groups were α-thalassemia and β-thalassemia, collectively accounting for about 76% to 79% of cases. Detailed SMDs before and after matching and the Love plot were provided in Supplementary Table S1 and Figure S1.

### 3.2. Cycle Characteristics and Embryological Outcomes

**Figure 1:**
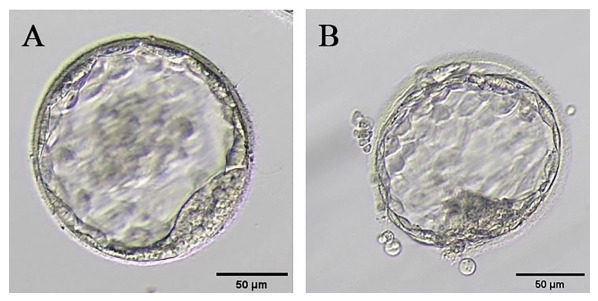
Representative anonymized embryology images from the Utrogestan-based PPOS and GnRH-ant groups. (**A**) Representative blastocyst from the Utrogestan-based PPOS group. (**B**) Representative blastocyst from the GnRH-ant group.

As shown in Table 2, the duration of Gn stimulation was comparable between groups, whereas the total Gn dosage was significantly lower in the Utrogestan-based PPOS group (2100 (1500–3000) vs. 2250 (1800–3000), *p =* 0.035). On the trigger day, estradiol (E_2_) levels and LH levels were similar in both groups. Progesterone (P) levels were significantly elevated in the Utrogestan-based PPOS (9.35 (7.32–14.01) ng/mL relative to the GnRH-Ant group (0.97 (0.68–1.36) ng/mL, *p* < 0.001), consistent with pharmacological characteristics. No significant differences were observed between groups in the number of oocytes retrieved, MII oocyte rate, 2PN fertilization rate, day-3 transferable embryo rate, blastocyst formation rate, blastocyst available-for-biopsy rate, or blastocysts available for biopsy rate per MII oocyte, although point estimates numerically favored the Utrogestan-based PPOS group for several embryologic outcomes. Representative anonymized embryology images are shown in Figure 1 and Figure S2.

### 3.3. PGT for Aneuploidy

In total, 1191 blastocysts underwent biopsy, including 586 from the Utrogestan-based PPOS protocol and 605 from the GnRH-Ant protocol, as summarized in Table 3. No statistically significant differences were detected between the two groups in euploidy rate (59.56% vs. 54.71%), aneuploidy rate (25.94% vs. 24.96%), mosaicism rate (14.16% vs. 19.67%), or “No-call” rate (0.34% vs. 0.66%). Importantly, the Utrogestan-based PPOS protocol showed a significantly higher unadjusted euploidy rate per MII oocyte (18.98% vs. 16.36%, *p* = 0.045). The distributions of single, double, and complex aneuploidy were comparable between the two groups.

To further address residual confounding, multivariable analyses were conducted, as summarized in Table 4. With the Utrogestan-based PPOS group as the reference, the GnRH-Ant group showed both lower euploid yield per MII oocyte and per biopsied embryo after adjustment for potential confounders; however, neither association reached statistical significance (B = −0.144, 95% CI −0.362 to 0.073, *p* = 0.194; and B = −0.196, 95% CI −0.446 to 0.053, *p* = 0.122, respectively). Among the covariates, total Gn dose was positively associated with euploid yield per MII oocyte (B = 0.000, 95% CI 0.000 to 0.000, *p* = 0.036), whereas AFC was independently associated with euploidy rate per biopsied embryo (B = 0.027, 95% CI 0.001 to 0.052, *p* = 0.041).

**Figure 2:**
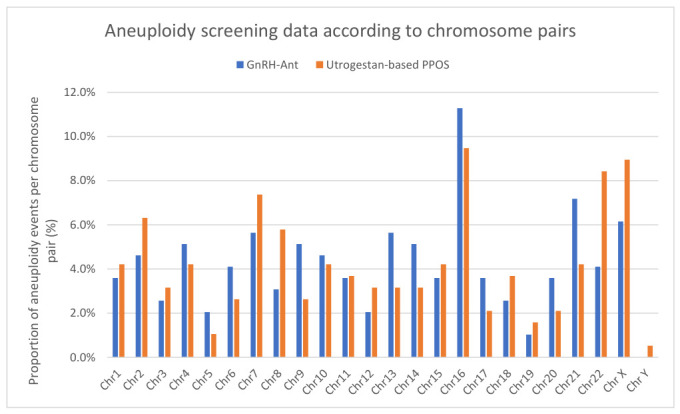
Distribution of chromosome-specific aneuploidy events. The *y*-axis represents the proportion of aneuploidy events attributable to each chromosome among all aneuploidy events, calculated as (events for a given chromosome/total aneuploidy events) × 100%. Data were derived from 303 blastocysts. Abbreviations: PPOS: progestin-primed ovarian stimulation; GnRH-Ant: gonadotropin-releasing hormone antagonist; Chr: chromosome.

To assess whether the Utrogestan-based PPOS protocol was associated with differences in chromosomal aberration patterns, aneuploidy data were summarized in Figure 2. Overall, chromosome 16 exhibited the highest frequency of aneuploidy events in both groups. The distribution of chromosomal abnormalities, including both autosomes and sex chromosomes, did not differ significantly between the two groups (*p* = 0.793).

### 3.4. Pregnancy Outcomes

Pregnancy outcomes were analyzed per first transfer cycle in patients with completed live-birth follow-up. The Utrogestan-based PPOS group showed numerically higher biochemical pregnancy, clinical pregnancy, and live birth rate, as well as a lower first-trimester pregnancy loss rate, than the GnRH-Ant group, though none of these differences reached statistical significance. As shown in Table 5, no significant differences were observed between the two groups in gestational age, birth weight, birth length, or low-birth-weight infant rate. Notably, one patient in the Utrogestan-based PPOS group underwent elective termination of pregnancy at 19 weeks of gestation because of congenital cardiac malformation. All prenatal diagnostic results were concordant with the corresponding PGT results.

## 4. Discussion

The potential effect of Utrogestan-based PPOS protocol on the embryonic developmental potential and chromosomal euploidy rate remains unclear due to limited evidence. To our knowledge, few studies have evaluated euploidy rate and chromosomal aberration patterns following Utrogestan-based PPOS within a low-confounding model—a PGT-M patient population. In this retrospective study, we demonstrated a comparable euploidy rate and a significantly lower total Gn dose in the Utrogestan-based PPOS protocol, as compared with the GnRH-Ant protocol. Moreover, the two protocols showed similar aneuploidy subtypes, analogous spectra of chromosomal abnormalities, and similar pregnancy outcomes. Utrogestan-based PPOS protocol may be a feasible alternative for ovarian stimulation in PGT-M cycles.

As summarized by Ata et al., several types of progestins can effectively suppress premature LH surge [16]. However, most published data focus on MPA- or dydrogesterone-based PPOS protocols, whereas evidence regarding oral micronized progesterone remains limited. Zhu et al. first reported in 2015 that Utrogestan-based PPOS achieved effective LH suppression and comparable reproductive outcomes to those of the short protocol [17]. Subsequently, the same group conducted a randomized controlled trial in polycystic ovary syndrome patients, demonstrating significantly higher fertilization and implantation rates in Utrogestan-based PPOS versus the short protocol [18]. Guo et al. also reported a significantly higher blastocyst formation rate in the Utrogestan-based PPOS group than in the MPA-based PPOS group [19]. In line with previous studies, our study showed Utrogestan-based PPOS required a statistically significantly lower total Gn dose while achieving comparable embryological outcomes. Therefore, the Utrogestan-based PPOS protocol was not associated with worse ovarian response or embryological outcomes.

The impact of the PPOS protocols on embryo euploidy rates remains controversial, with conflicting findings across current studies. Most investigations have shown that the euploidy rate of embryos derived from MPA-based PPOS protocol is comparable to that observed with conventional ovarian stimulation regimens [20,21], although findings from age-stratified analyses remain inconsistent [22,23]. Studies on dydrogesterone-based protocols have similarly reported a similar euploidy rate between dydrogesterone-based PPOS and GnRH-Ant protocols [11,12,24]. In our cohort, the overall euploidy rate per biopsied blastocyst was comparable between the Utrogestan-based PPOS protocol and GnRH-Ant protocol. Although the unadjusted analysis suggested a numerically higher euploid yield per MII oocyte in the Utrogestan-based PPOS group, this difference did not persist after multivariable adjustment. Thus, the apparent unadjusted advantage may have been influenced by residual confounding or other baseline differences, and no independent association between the Utrogestan-based PPOS protocol and euploidy outcomes was observed.

To the best of our knowledge, few studies have reported the effect of PPOS protocols on chromosomal aneuploidy subtypes or the distribution of aneuploid events across individual chromosomes to date. In our study, both protocols exhibited similar patterns in terms of chromosomal aneuploidy subtypes and the distribution of involved chromosomes. These descriptive findings were consistent with the absence of a major difference in ploidy patterns between the two protocols. However, given the limited sample size, the observed similarity should be interpreted with caution and does not exclude the possibility of subtle differences or effects on protocol-specific effects on individual chromosomes. Future mechanistic studies are needed to investigate whether progesterone exposure has any direct influence on chromosomal segregation.

Consistent with previous findings [25,26], pregnancy outcomes were comparable between the two groups. Embryo developmental potential also appeared to be similar between the Utrogestan-based PPOS and antagonist protocols. Regarding congenital malformations, Liang Zhou et al. reported that there were no significant differences in neonatal outcomes or congenital malformations among PPOS, GnRH-antagonist, and GnRH-agonist protocols [27]. In this cohort, only one case of termination of pregnancy because of congenital heart disease was observed in the Utrogestan-based PPOS group, whereas no congenital malformations were found in the GnRH-Ant group. However, given the limited number of clinical pregnancies (n = 53) and live births (n = 47), the current study was not adequately powered to evaluate the safety of the Utrogestan-based PPOS protocol with respect to congenital anomalies. Therefore, no conclusions regarding the risk of congenital malformations can be drawn from these data. Future studies with larger cohorts and dedicated long-term follow-up are warranted to address this important safety issue.

This study has several strengths. First, the matched design and multivariable analyses enhanced the reliability of the findings by reducing the influence of confounders. Second, the use of a relatively homogeneous PGT-M population reduces the heterogeneity commonly observed in PGT-A cohorts. Furthermore, to our knowledge, this study is among the first to report comparable patterns of chromosomal aneuploidy between Utrogestan-based PPOS and GnRH-Ant protocols.

Several limitations should also be acknowledged. First, the retrospective nature and limited sample size may have introduced potential confounding factors and bias. Second, the sample size was determined by the number of eligible patients during the study period without a priori power calculation. The post hoc powers calculated for euploidy rate per biopsied embryo and live birth rate were 29.9% and 57.0%, respectively, indicating limited statistical power for these outcomes. Therefore, the non-significant findings, particularly those regarding the euploidy rate per biopsied embryo and live birth rate, should not be interpreted as evidence of equivalence or absence of a true between-group difference. Instead, these results should be considered exploratory and interpreted with caution, given the increased possibility of type II errors. Future prospective studies with larger sample sizes are warranted to confirm the observed trends. Third, although the relatively homogeneous PGT-M population was deliberately chosen to minimize confounding by infertility etiology, this same homogeneity limits the generalizability of our findings to broader IVF populations, including routine IVF, PGT-A, recurrent implantation failure, or recurrent miscarriage populations. Fourth, cumulative live birth rate was not assessed because follow-up data on subsequent frozen-thawed embryo transfer cycles were incomplete, which is a limitation inherent to the retrospective design. The long-term safety of the Utrogestan-based PPOS regimen requires further validation through extended follow-up studies, with particular attention to critical endpoints, including cumulative live birth rates, neonatal birth defects, and subsequent growth and development outcomes.

## 5. Conclusions

In this retrospective matched cohort study, the Utrogestan-based PPOS protocol achieved comparable embryological, chromosomal, and pregnancy outcomes to the GnRH-Ant protocol, while requiring lower gonadotropin consumption. These findings suggest that the Utrogestan-based PPOS protocol may represent a feasible alternative ovarian stimulation option in PGT-M cycles. However, given the retrospective design, limited statistical power, and small number of live births, larger prospective multicenter studies are required before definitive conclusions regarding its safety and effectiveness can be drawn.

## Figures and Tables

**Table 1:** Baseline characteristics of eligible patients.

Items	Utrogestan-Based PPOS	GnRH-Ant	SMDs	*p* Value ^1^
No. of cycles	176	176		
Maternal age (years)	31.57 ± 3.77	31.45 ± 3.79	0.03	0.778
Paternal age (years)	33.03 ± 4.21	33.49 ± 4.87	0.101	0.344
BMI (kg/m^2^)	21.55 (19.3–24)	21.3 (19.6–23.9)	0.037	0.988
AMH (ng/mL)	3.04 (1.7–4.85)	3.06 (1.7–4.78)	0.002	0.989
AFC	13 (9–16.75)	13 (9–17)	−0.020	0.852
Basal FSH (IU/L)	6.62 (5.56–8.03)	6.61 (5.63–7.89)	−0.056	0.873
Basal E_2_ (pg/mL)	36 (28.33–47)	36 (29.95–48.18)	0.019	0.691
Basal LH (IU/L)	5.1 (3.88–6.47)	5.3 (4.23–6.61)	−0.087	0.199
Indication or PGT-M, *n* (%)				0.771
α-thalassemia	95 (53.98%)	103 (58.52%)		
β-thalassemia	39 (22.16%)	36 (20.45%)		
other monogenic diseases	42 (23.86%)	37 (21.02%)		

^1^ *p* Values for continuous variables were calculated using paired Student’s *t*-tests or Wilcoxon signed-rank tests and for categorical variables using Bowker’s tests. Abbreviations: PPOS: progestin-primed ovarian stimulation; GnRH-Ant: gonadotropin-releasing hormone antagonist; SMD: standardized mean difference; PGT-M: preimplantation genetic testing for monogenic disorders; BMI: body mass index; AMH: anti-Müllerian hormone; AFC: antral follicle count; FSH: follicle-stimulating hormone; E_2_: estradiol; LH: luteinizing hormone.

**Table 2:** Cycle characteristics and embryological outcomes between Utrogestan-based PPOS and GnRH-Ant protocols.

Items	Utrogestan-Based PPOS	GnRH-Ant	*p* Value ^1^
Total Gn dose (IU)	2100 (1500–3000)	2250 (1800–3000)	0.035
Gn days (d)	9 (8–10)	9 (8–10)	0.609
E_2_ on trigger day (pg/mL)	3029 (1839.25–5417.5)	2928.5 (1548.75–4553.75)	0.12
LH on trigger day (IU/L)	2.28 (1.28–3.52)	2.35 (1.42–4.2)	0.426
P on trigger day (ng/mL)	9.35 (7.32–14.01)	0.97 (0.68–1.36)	<0.001
Total No. of oocytes retrieved	2381	2578	
No. of oocytes retrieved	13 (7–18)	14 (8–19)	0.273
MII rate, *n* (%)	1839 (77.24%)	2023 (78.47%)	0.440
2PN rate, *n* (%)	1432 (77.87%)	1566 (77.41%)	0.925
D3 transferable embryo rate, *n* (%)	1075 (75.07%)	1134 (72.41%)	0.380
Blastocyst formation rate, *n* (%)	817 (75.72%)	829 (72.34%)	0.213
Blastocyst available for biopsy formation rate, *n* (%)	586 (54.31%)	605 (52.79%)	0.508
Blastocyst available for biopsy rate per MII oocyte, *n* (%)	586 (31.87%)	605 (29.91%)	0.395

^1^ *p* values for continuous variables were calculated using paired Wilcoxon signed-rank tests, and Proportional embryological outcomes were compared using GEE with a binomial distribution and logit link function. Abbreviations: PPOS: progestin-primed ovarian stimulation; GnRH-Ant: gonadotropin-releasing hormone antagonist; Gn: gonadotropin; E_2_: estradiol; LH: luteinizing hormone; P: progesterone; No.: number; MII: metaphase II; 2PN: two pronuclei.

**Table 3:** Comparison of PGT-A results between groups.

Items	Utrogestan-Based PPOS	GnRH-Ant	*p* Value ^1^
No. of embryos for biopsy	586	605	
PGT-A results, *n* (%)			0.124
Euploidy rate per biopsied embryo ^2^	349 (59.56%)	331 (54.71%)	
Aneuploidy rate per biopsied embryo	152 (25.94%)	151 (24.96%)	
Mosaicism rate per biopsied embryo	83 (14.16%)	119 (19.67%)	
“No-call” rate per biopsied embryo	2 (0.34%)	4 (0.66%)	
Aneuploidy types, *n* (%)			0.579
Single-chromosome aneuploidy rate	97 (63.82%)	89 (58.94%)	
Double-chromosomes aneuploidy rate	36 (23.68%)	39 (25.83%)	
Complex chromosomes abnormal aneuploidy rate	19 (12.50%)	23 (15.23%)	
Euploidy rate per oocyte retrieved, *n* (%)	349 (14.66%)	331 (12.84%)	0.256
Euploidy rate per MII oocyte, *n* (%)	349 (18.98%)	331 (16.36%)	0.045

^1^ *p*-values for the distribution of PGT-A outcomes were calculated using cluster-robust multinomial logistic regression, and proportional embryological outcomes were compared using GEE. ^2^ Post hoc power was calculated for euploidy rate per biopsied embryo the using observed event rates and sample sizes (two-tailed α = 0.05): 29.9%. Euploidy rate per oocyte retrieved was calculated as (number of euploid embryos/total number of oocytes retrieved) × 100%. Euploidy rate per MII oocyte was calculated as (number of euploid embryos/total number of MII oocytes) × 100%. Abbreviations: PPOS: progestin-primed ovarian stimulation; GnRH-Ant: gonadotropin-releasing hormone antagonist; PGT-A: preimplantation genetic testing for aneuploidy; MII: metaphase II.

**Table 4:** Multivariable analysis for euploid yield per MII oocyte and euploidy rate per biopsied embryo.

Variables	Euploid Yield per MII Oocyte			Euploidy Rate per Biopsied Embryo		
	B	95% CI	*p* Value	B	95% CI	*p* Value ^1^
GnRH-Ant vs. PPOS group ^a^	−0.144	−0.362 to 0.073	0.194	−0.196	−0.446 to 0.053	0.122
Female age	0.005	−0.025 to 0.035	0.747	0.003	−0.036 to 0.041	0.883
AMH (ng/mL)	−0.073	−0.141 to 0.035	0.079	−0.009	−0.077 to 0.058	0.791
AFC	0.016	−0.006 to 0.039	0.156	0.027	0.001 to 0.052	0.041
BMI (kg/m^2^)	0.026	−0.006 to 0.058	0.115	0.025	−0.001 to 0.065	0.210
Total Gn dose (IU)	0.000 ^b^	0.000 to 0.000	0.036	4.813 × 10^−5^	0.000 to 0.000	0.703
Gn days (d)	0.031	−0.097 to 0.160	0.631	−0.008	−0.131 to 0.115	0.904

^1^ *p*-values for multivariable analyses were calculated using GEEs with a binomial distribution and logit link function. ^a^ PPOS group was used as the reference group in both models. ^b^ The coefficient was positive but very small and was rounded to 0.000 in the SPSS output. Abbreviations: MII: metaphase II; GnRH-Ant: gonadotropin-releasing hormone antagonist; PPOS: progestin-primed ovarian stimulation; AMH: anti-Müllerian hormone; AFC: antral follicle count; BMI: body mass index; CI: confidence interval; Gn: gonadotropin.

**Table 5:** Comparison of pregnancy outcomes of FETs between groups.

Items	Utrogestan-Based PPOS	GnRH-Ant	*p* Value ^1^
No. of transfer cycle	75	87	
Biochemical pregnancy rate, *n* (%)	59 (78.67%)	63 (72.41%)	0.328
Clinical pregnancy rate, *n* (%)	53 (70.67%)	55 (63.22%)	0.320
Ectopic pregnancy rate, *n* (%)	1 (1.89%)	1 (1.82%)	NA ^3^
Pregnancy loss in first trimester, *n* (%)	3 (5.66%)	7 (12.73%)	0.113
Pregnancy loss in second or third trimester, *n* (%)	1 (1.89%)	1 (1.82%)	NA ^3^
Pregnancy terminated artificially in second or third trimester because of defect, *n* (%)	1 (1.89%)	0 (0)	NA ^3^
Stillbirth rate, *n* (%)	0 (0)	1 (1.82%)	NA ^3^
Live birth rate ^2^, *n* (%)	47 (62.67%)	45 (51.72%)	0.189
Gestational age, week	37.94 ± 2.08	37.87 ± 1.92	0.868
Birth weight, g	3248.32 ± 450.38	3076 ± 511.18	0.09
Birth length, cm	49.52 ± 2.15	49.17 ± 2.68	0.499
Low-birth-weight infant rate, *n* (%)	3 (6.38%)	3 (6.67%)	1

^1^ *p* Values for continuous variables were calculated using paired Student’s *t*-tests, and proportional outcomes were compared using generalized estimating equations (GEE). ^2^ Post hoc power for the live birth rate was calculated using observed event rates and sample sizes (two-tailed α = 0.05): 57.0%. ^3^ Due to low event frequency, these data are presented descriptively; no *p* value was calculated. Abbreviations: PPOS: progestin-primed ovarian stimulation; GnRH-Ant: gonadotropin-releasing hormone antagonist; - FET: frozen-thawed embryo transfer.

## Data Availability

The original contributions presented in this study are included in the article. Further inquiries can be directed to the corresponding authors.

## Supplementary Materials

The following supporting information can be downloaded at https://www.mdpi.com/article/10.3390/biomedicines14071629/s1, Table S1. Balance diagnostics before and after direct caliper matching. Figure S1. Love plot of covariates. Figure S2. Additional representative anonymized embryology images from the Utrogestan-based PPOS and GnRH-ant groups. (a–c) representative blastocyst from Utrogestan-based PPOS group. (d–f) representative blastocyst from GnRH-ant group. No patient-related information, including names, medical record numbers, cycle identifiers, dates, or embryo identifiers, is displayed.

## Abbreviations

The following abbreviations are used in this manuscript:

COH	controlled ovarian hyperstimulation
PPOS	progesterone-primed ovarian stimulation
GnRH-Ant	gonadotropin-releasing hormone antagonist
PGT-M	preimplantation genetic testing for monogenic disorders
AMH	anti-Müllerian hormone
AFC	antral follicle count
BMI	Body Mass Index
Gn	gonadotropin
MII	metaphase II
2PN	two pronuclei
LH	luteinizing hormone
MPA	medroxyprogesterone acetate
PGT-A	preimplantation genetic testing for aneuploidy
FET	frozen-thawed embryo transfer
SNP	single-nucleotide polymorphism
MAPD	Median of the Absolute Pairwise Difference
CV	coefficient of variation
QC	quality control
SD	standard deviation
IQR	interquartile range
GEE	generalized estimating equations
FSH	follicle-stimulating hormone
E2	estradiol
P	progesterone
No.	number
Chr	chromosome
