# Supplementary material for: Comparable Euploidy and Aneuploidy Patterns Between Utrogestan-Based Progesterone-Primed Ovarian Stimulation and GnRH Antagonist in PGT-M: A Retrospective Matched Cohort Study
Source: Biomedicines. 2026 Jul 20;14(7):1629. doi: 10.3390/biomedicines14071629 (PMC13406216; doi:10.3390/biomedicines14071629)
Supplement: Supplementary file 1 [file biomedicines-14-01629-s001.zip › biomedicines-4317443-supplementary.pdf]

Supplementary Table S1. Balance diagnostics before and after direct caliper matching.

Variable	Before matching SMD	After matching SMD
Maternal age (years)	0.371	0.03
Parental age (years)	0.244	0.101
BMI (kg/m^2)	0.123	0.037
AMH (ng/ml)	-0.537	0.002
AFC	-0.282	-0.02
Basal FSH (IU/L)	0.176	-0.056
Basal E2 (pg/mL)	0.097	0.019
Basal LH (IU/L)	-0.35	-0.087

Abbreviations: SMD: standardized mean differences, BMI: Body Mass Index, AMH: anti-Mü llerian hormone, AFC: antral follicle count, FSH: follicle-stimulating hormone, E2: estradiol, LH: luteinizing hormone

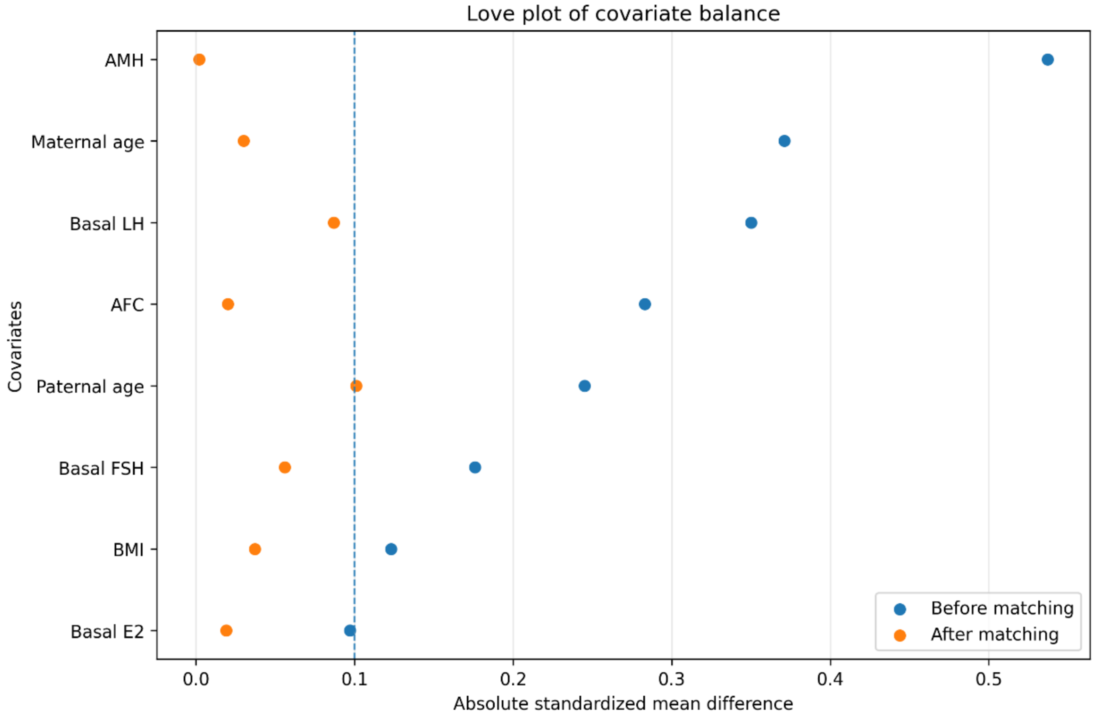

Supplementary FigureS1. Love plot of covariates.

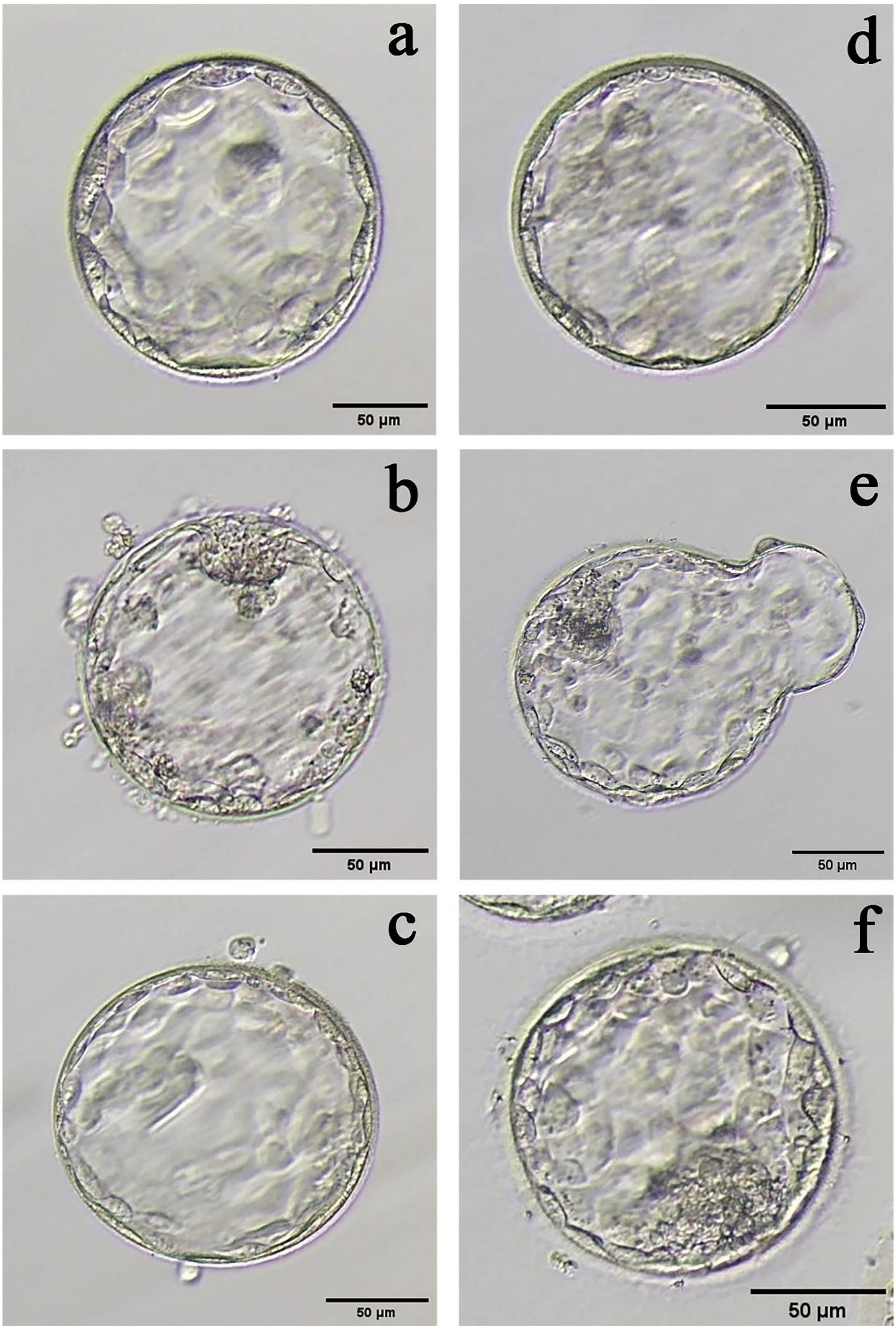

Supplementary Figure S2. Additional representative anonymized embryology images from the Utrogestan-based PPOS and GnRH-ant groups. (a-c) representative blastocyst from Utrogestan-based PPOS group. (d-f) representative blastocyst from GnRH-ant group. No patient-related information, including names, medical record numbers, cycle identifiers, dates, or embryo identifiers, is displayed.
